# Revealing the mystery of the cases where Nd–Fe–B magnetic like poles attract each other

**DOI:** 10.1038/s41598-021-91969-8

**Published:** 2021-06-15

**Authors:** Hui Meng, Guiping Tang, Abby Shen, Michelle Qian, Qifeng Wei, George Mizzell, Christina H. Chen

**Affiliations:** 1 Foresee Group, Zhejiang, 311500 China; 2 Quadrant at Hangzhou, Zhejiang, 311500 China; 3 Quadrant at San Jose, San Jose, CA 95131 USA; 4 SuperMagnetMan, Pelham, AL 35124 USA

**Keywords:** Engineering, Materials science, Physics

## Abstract

This investigation reveals the mystery of the cases where magnetic like poles attract each other, and unlike poles repel one another. It is identified that for two unequally sized like poles, the pole with a higher *P*_*c*_ (permeance coefficient) causes a localized demagnetization (*LD*) to the pole with a lower *P*_*c*_. If the *LD* is large enough, the polarity of a localized area can be reversed, resulting in an attraction between these two like poles in the *LD* area in a small gap. Two unusual behaviors are observed: (1) an inflection point *IP* appears on the force vs gap curves of all the unequally sized like poles since they have different *P*_*c*_. Normally, the like poles’ repelling force increases when the gap decreases, but this *IP* results in nonmonotonic curves, even an attractive force in a small gap; (2) for some NdFeB magnets with a low coercivity and nonlinear B–H curve in the 2nd quadrant, a repulsion can occur for these unequal sized unlike poles, after previously pairing with their like poles that left an unrecoverable *LD* and reversed polarity area. The relationship of the *LD*, the *P*_*c*_ ratio, and the B–H curve are also explored in this paper.

## Introduction

The basic law of magnetism is that like poles repel one another, and unlike poles attract each other. Even though Gauss’ law for magnetic flux density (B-field) indicates that there is no free magnetic charge, we can still define the effective bound magnetic charges locally from the magnetization of magnetic material^1^. The distribution of positive magnetic charge can be defined as the “north pole”, and correspondingly, the negative magnetic charge can be defined as the “south pole”. The interaction between the local magnetic charges is governed by Coulomb’s law so that like charges repel and unlike charges attract^2^. However, George Mizzell observed some cases in which two like poles attracted each other near the central area for a pair of permanent magnets with significantly different dimensions. Mizzell first reported it in May 2007^3^ on YouTube, then reported it again in March 2019^4^. We reported our preliminary investigation for this seemingly unacceptable behaviour in November 2019^5^. Other researchers also reported such a fact in 2019^6^. The phenomenon does not mean that the fundamental laws of magnetism are violated, but it is important to understand the physics underlying the phenomenon. Why do these “like poles” attract each other instead of repelling? To understand the mechanism and reveal the mystery of this unique interaction, a series of experiments were conducted in our labs.

The permeance coefficient (*P*_*c*_) is defined as the ratio of magnetic induction *B*_*d*_ and magnetic field *H*_*d*_ inside a standalone magnet at the operating point, i.e., *P*_*c*_ =|*B*_*d*_*/H*_*d*_|^7,8^, which depends on the geometry of the magnet. For example, in the case of cylindrical magnets with the same diameter and same magnetization along the axis, the longer the magnet is, the higher the *P*_*c*_. Combined with the B–H curves, the *P*_*c*_ can determine how easily a magnet will be demagnetized, especially when the B–H curve in the 2nd quadrant is nonlinear. In this work, we find that the *P*_*c*_ and the B–H curves are the key factors to explain the interesting phenomena.

## Experiment method

**Figure 1:**
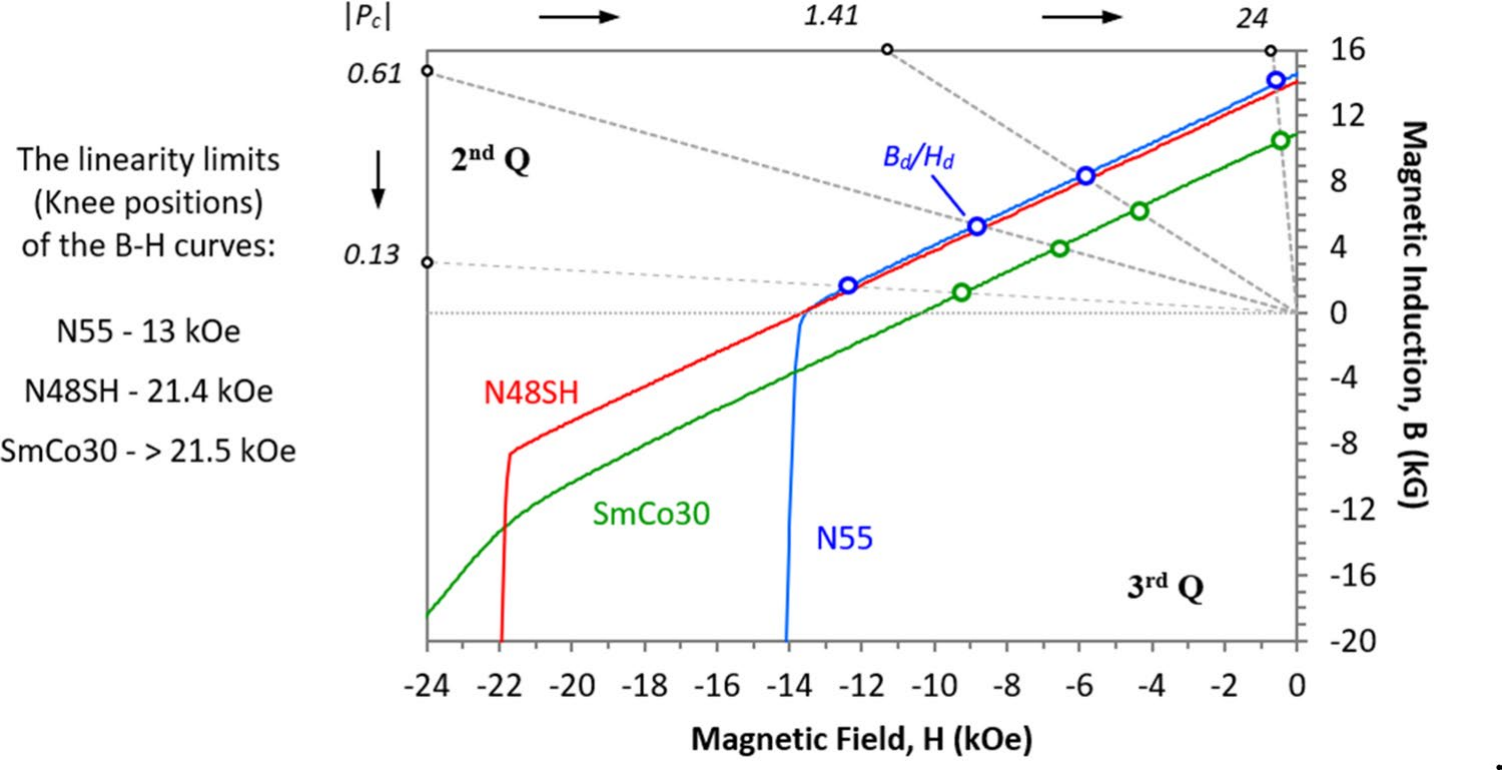
The B–H curves of the three magnets in the 2nd and part of the 3rd quadrant, and the working points *B*_*d*_*/H*_*d*_ marked for N55 and SmCo30 for four load lines with *P*_*c*_ = 0.13–24.

N55 and N48SH samples of Nd–Fe–B, and SmCo30 samples of Sm_2_Co_17_ were tested. The dimensions and values of *P*_*c*_ are shown in Table 1, and their demagnetization B–H curves are shown in Fig. 1. These cylindrical magnet pairs with various *P*_*c*_ values range from 0.13 to 24. For the material itself, N55 has a nonlinear B–H curve in the 2nd quadrant, while N48SH and SmCo30 have linear B–H curves in the 2nd and even in part of the 3rd quadrant. SmCo30 was tested so that the surface degradation can be excluded, which may be more obvious for some small or thin Nd–Fe–B magnets^9^.

**Table 1:** The magnet samples tested in this investigation (the unit is mm, where OD and L are the diameter and length of the cylinders, respectively).

Pair ID	Magnet 1 (top)			Magnet 2 (bottom)			N55	N48SH	SmCo30
	*OD*	*L*	*P*_*c1*_	*OD*	*L*	*P*_*c2*_			
1-A	4	2	1.41	32	2	0.13	✓	✓	✓
2-A	8	2	0.61				✓	✓	✓
3-A	16	2	0.28				✓	✓	
A-A	32	2	0.13				✓		✓
4-A	4	16	24	32	2	0.13	✓	✓	✓
4-B				32	4	0.28	✓	✓	
4-C				32	8	0.61	✓	✓	
4-D				32	16	1.41	✓	✓	✓

**Figure 2:**
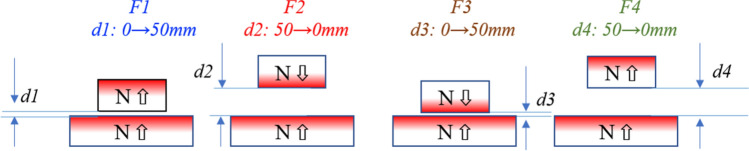
Four forces testing setup: *F*_*1*_*, F*_*2*_*, F*_*3*_, and *F*_*4*_ as the above sequence. *F*_*1*_ and *F*_*4*_ should be all attracting and *F*_*2*_ and *F*_*3*_ should be all repelling, and “*d”* is the gap in between*.*

**Figure 3:**
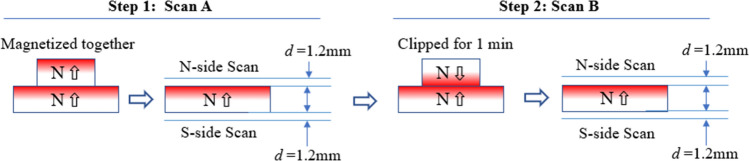
Surface flux testing setup: scan on the surface with 0.5 mm intervals, and gap *d* is 1.2 mm between the Hall sensor and the surface due to Hall probe construction and the clearance.

These magnet pairs were tested for repelling and attracting forces at the gaps between 0 and 50 mm at the center position, using an Instron 5944 force tester. In order to observe what happened during the test process, four forces were recorded and marked by their testing sequence, *F*_*1*_, *F*_*2*_*, F*_*3*_, and *F*_*4*_, as shown in Fig. 2. *F*_*1*_ and *F*_*4*_ were recorded for N **→ **S unlike poles, and *F*_*2*_ and *F*_*3*_ for N **→← **N like poles. In addition to the force test, some of the magnets with OD = 32 mm were also tested for flux density on the surface to estimate the level of the localized demagnetization *LD*, using a Brockhaus XYZ Scanner. The Hall sensor was 1.2 mm above the magnet surface due to the Hall probe construction and the clearance, and this gap was maintained throughout the experiment. There were two steps in sequence to test the surface flux, as shown in Fig. 3. Step 1, the pairs were magnetized together aligning at the center, then scanned the surfaces of both sides N and S on the bottom magnet after the top magnet was removed; Step 2, turned the top magnet upside down and touched the bottom magnet at the center for 1 min, then separated, scanned the bottom magnet at the surface for both sides N and S.

## Results and analysis

### Unusual behaviours shown in *F*_*2*_ and *F*_*4*_

**Figure 4:**
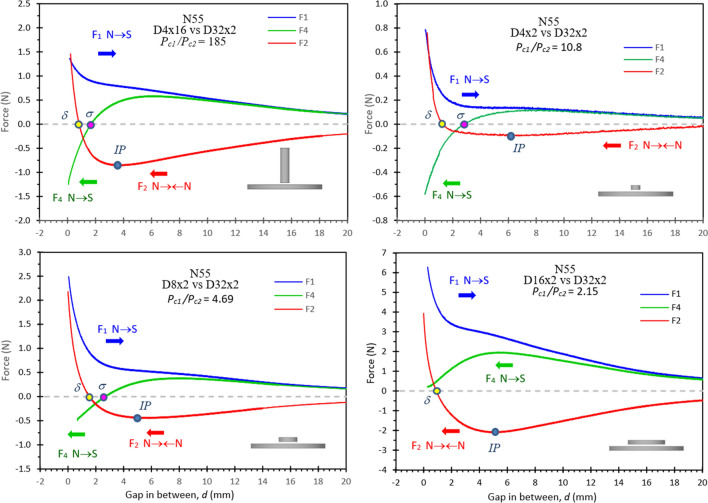
Forces vs gap for some unequally sized N55 pairs, showing the following: (1) an inflection point *IP* appears on the curve of *F*_*2*_ vs *d*; (2) *F*_*4*_ < *F*_*1*_ or even *F*_*4*_ < 0 when *d* < *σ*, and *F*_*2*_ > *0 when d* < *δ*. (**a**) *F*_*3*_ is not plotted here as *F*_*3*_ ≅ *F*_*2*_. (**b**) δ is the point where *F*_*2*_ = 0, and σ is the point where *F*_*4*_ = 0.

Figure 4 plots *F*_*1*_, *F*_*2*_, and *F*_*4*_ forces vs. the gap *d* for some N55 unequally sized pairs. (*F*_*3*_ is not plotted here as *F*_*3*_ is the same as *F*_*2*_). Two unusual behaviours are observed in the plots: (1) an inflection point *IP* appears on the curve of *F*_*2*_ vs *d* for the N **→← **N pairs of like poles. Normally, *F*_*2*_ is a repelling force (*F*_*2*_ < 0), and |*F*_*2*_| increases when the gap decreases, but this *IP* results in nonmonotonic curves, even attracting force. When the gap reduces to *d* < *IP*, |*F*_*2*_| starts to decrease and eventually transforms from repulsion to attraction (*F*_*2*_ > 0) at gap *d* < *δ* (~1 mm). It should be noted that the *IP* is caused by a localized demagnetization *LD,* which starts playing its effective role well before the *IP* point. (2) For some NdFeB magnets with a low coercivity and nonlinear B–H curve in the 2nd quadrant, repulsion can occur for these unequally sized unlike poles, after previously pairing with their like poles that left an unrecoverable *LD* and reversed polarity area. A force difference ∆*F* occurs to the unlike pole pairs, testing before and after pairing with their like poles. *F*_*4*_ < *F*_*1*_ and even *F*_*4*_ < *0* are observed for these N55 N **→ **S pairs. Normally, *F*_*1*_ and *F*_*4*_ have the same attracting force (*F*_*1*_ = *F*_*4*_ > *0*), as they were tested on the same N **→ **S pairs. The only difference is that *F*_*4*_ was tested after *F*_*2*_, and *F*_*2*_ was tested with N **→← **N like poles, which experienced the *LD*. When gap *d* reduces to a range (~ 15 to 8 mm), where the *LD* starts playing its role to cause an unusual effect, an unusual behaviour of *F*_*4*_ < *F*_*1*_ occurs. Three pairs with *P*_*c1*_*/P*_*c2*_ = 4.69, 10.8 and 185 have *F*_*4*_ < *0* when the gap *d* < *σ* (~ 2–3 mm), where the force transforms from attraction to repulsion, and only one pair (*P*_*c1*_*/P*_*c2*_ = 2.15) does not have *F*_*4*_ < *0*. This unusual *F*_*4*_ < *F*_*1*_ does not occur to N48SH and SmCo30, which have linear B–H curves.

**Figure 5:**
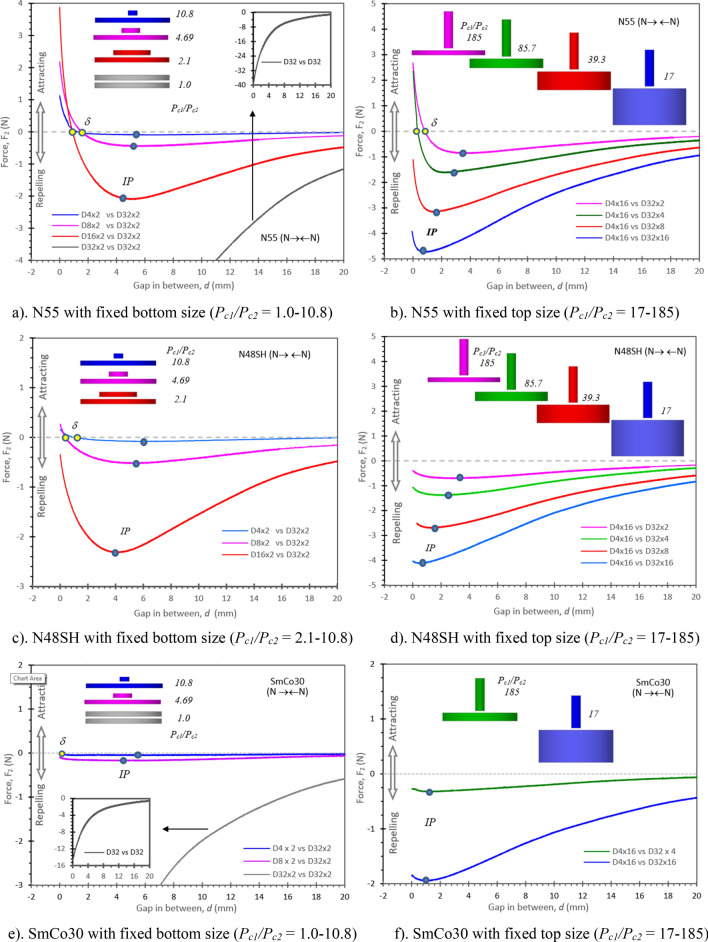
*F*_*2*_ vs gap *d* for N55, N48SH, and SmCo30 with both unequally sized and equally sized pairs. (**a**) *IP* is the inflection point. (**b**) *δ* is the point where *F*_*2*_ = 0.

Figure 5 shows *F*_*2*_ vs *d* curves of the N **→← **N pairs for N55, N48SH and SmCo30, with both unequally sized and equally sized pairs. An unusual *IP* is observed for all the magnet pairs, except the equally sized pairs with *P*_*c1*_ = *P*_*c2*_. Figure 5a,b for N55 show five out of eight pairs having *F*_*2*_ > *0* at *d* < *δ* (~1 mm). Figure 5c,d for N48SH display two pairs having *F*_*2*_ > *0* at *d* < *δ*. Figure 5e,f for SmCo30 present one pair (*P*_*c1*_/*P*_*c2*_ = 10.8) with *F*_*2*_ = *0* at *d* = *δ* (0.05 mm), which indicates that certain SmCo30 pairs can also have *F*_*2*_ > *0* at a small gap. It is noticeable in Fig. 5 that the higher the *P*_*c*_ ratio is, the higher the *IP* will be, and the *IP* ranges from 0.4 to 6 mm when *P*_*c1*_/*P*_*c2*_ is 2.1 to 185. When *P*_*c1*_/*P*_*c2*_ = 1 for the equal sized pairs, the *IP* disappears (or *IP* → 0).

**Figure 6:**
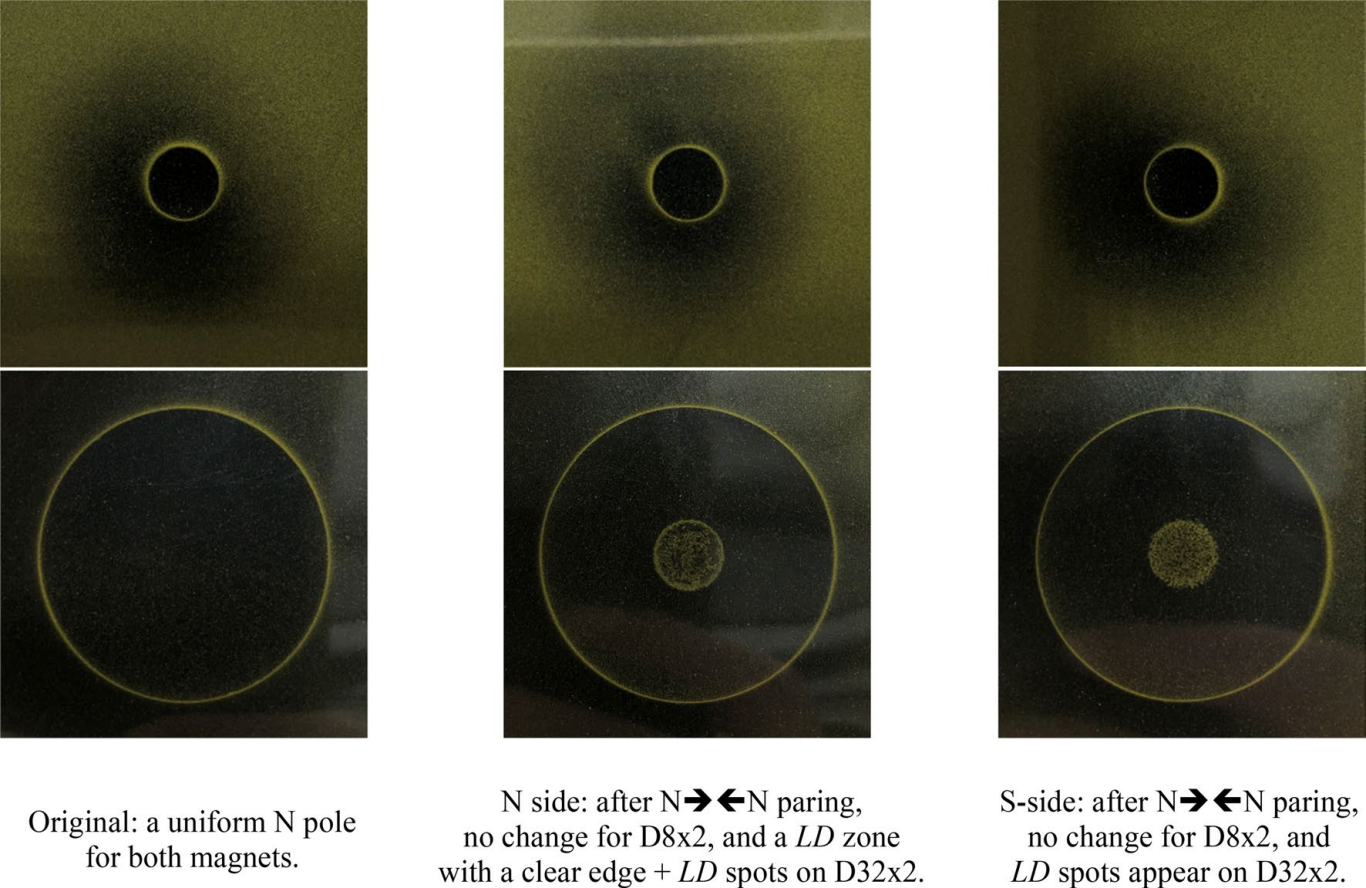
The green-tape views for N55 pair of D8 × 2 and D32 × 2 (*P*_*c*_ ratio = 4.69) before and after N **→← **N paring.

Figure 6 shows the green tape views for the magnets with  a *P*_*c*_ ratio of 4.69, before and after the N **→← **N paring. The reversed polarity is clearly seen on the D32 × 2 magnet after the paring, and no change is observed for the D8 × 2 magnet.

**Figure 7:**
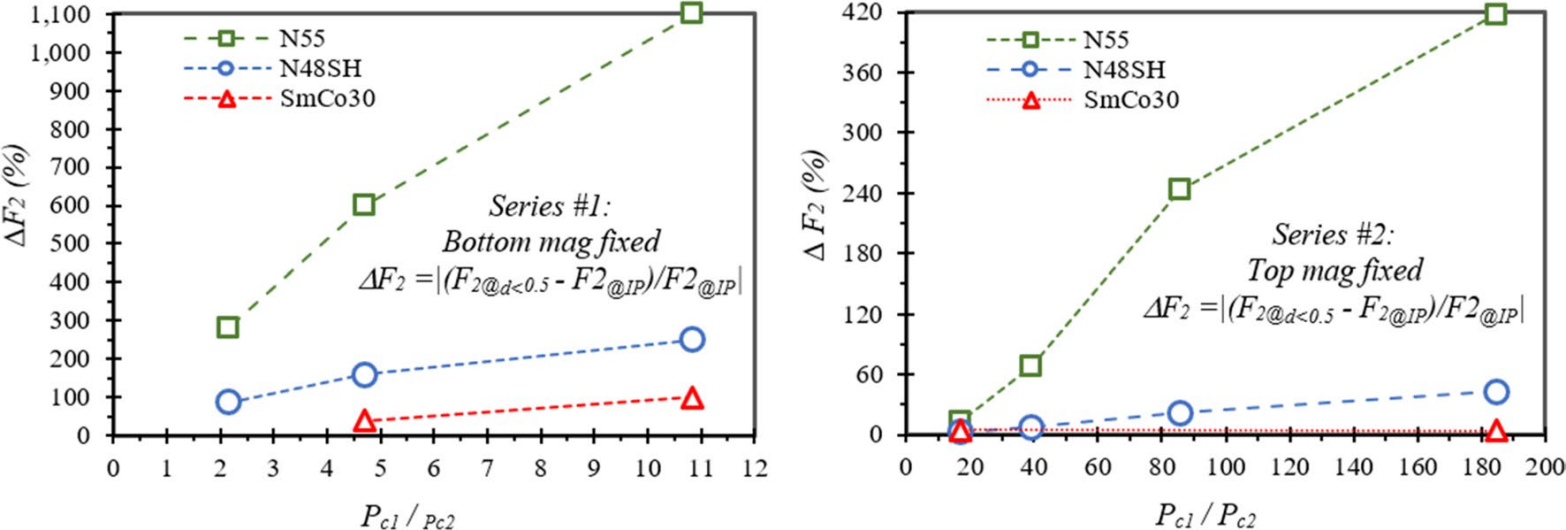
Unusual *ΔF*_*2*_ for all the magnet pairs with *P*_*c*1_ ≠ *P*_*c2*_: A higher *P*_*c1*_*/P*_*c2*_ leads to a higher *ΔF*_*2*_. All the pairs with *ΔF*_*2*_ > 100% show attraction for N **→← **N pairs.

**Figure 8:**
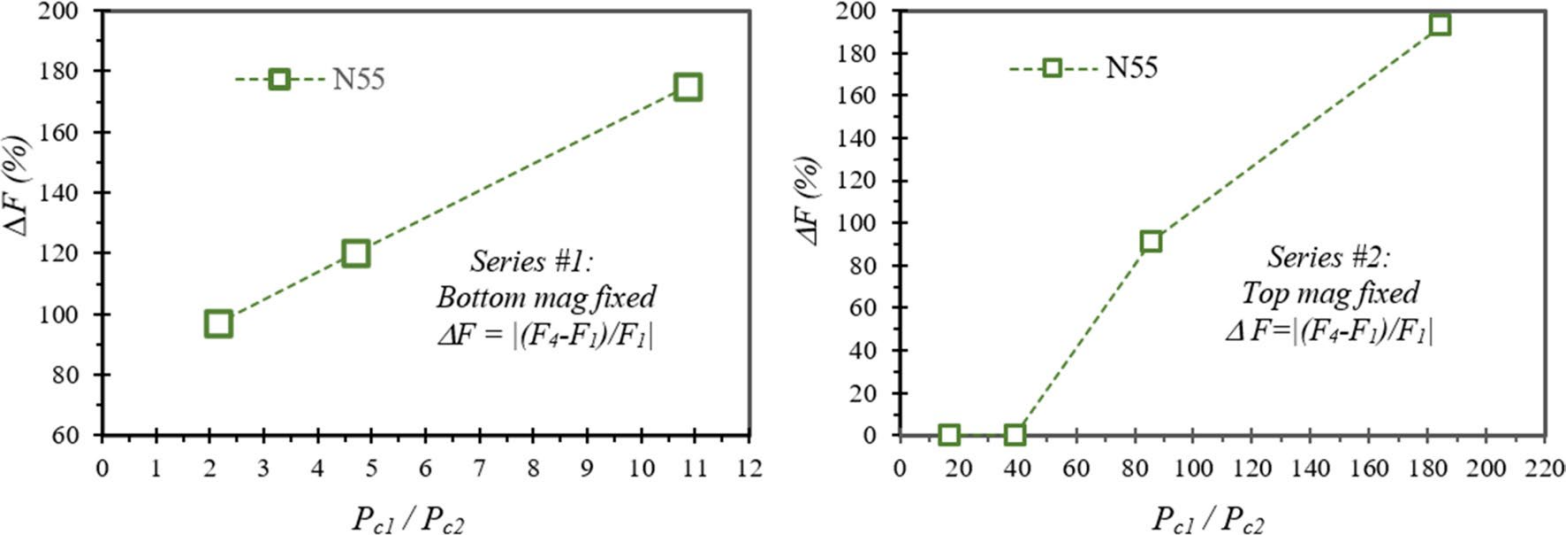
Unusual *F*_*4*_ < *F*_*1*_ for the N55 pairs with *P*_*c*1_ ≠ *P*_*c2*_: a higher *P*_*c1*_*/P*_*c2*_ results in a larger *ΔF*. Note: All the pairs with *ΔF* > 100% show repulsion for N **→ **S pairs.

Most of the results are listed in Table 2, from which the unusual behaviours of *F*_*2*_ and *F*_*4*_ can be analysed, and the localized demagnetization *LD* can be estimated. The data in Table 2 are plotted in Figs. 7 and 8 to make reasonable analyses. Figure 7 shows the force difference *ΔF*_*2*_ (= |(*F*_*2@d*<*0.5*_ − *F*_*2@IP*_)/*F*_*2@IP*_|) vs *P*_*c1*_*/P*_*c2*_. In general, a higher *P*_*c*_ ratio results in a larger *ΔF*_*2*_, which is as high as 1100% for N55 with *P*_*c1*_*/P*_*c2*_ = 10.8 in Series #1, and 400% for N55 with *P*_*c1*_*/P*_*c2*_ = 185 in Series #2. The *ΔF*_*2*_ is 2–250% for N48SH pairs, and 4–100% for SmCo30 pairs. Figure 8 demonstrates the force difference *ΔF* (= |(*F*_*4*_ − *F*_*1*_)/*F*_*1*_|) vs *P*_*c1*_*/P*_*c2*_ for N55 pairs only, as N48SH and SmCo30 do not have this unusual behaviour of *F*_*4*_ < *F*_*1*_. It is clear that a higher *P*_*c*_ ratio results in a larger *ΔF* for both Series #1 with the bottom size fixed and Series #2 with the top size fixed. The *ΔF* is as high as 193% for *P*_*c1*_*/P*_*c2*_ = 185 in Series #2, and 175% for *P*_*c1*_*/P*_*c2*_ = 10.8 in Series #1. A larger *ΔF* or *ΔF*_*2*_ indicates a greater localized demagnetization *LD*.

**Table 2:** Unusual behaviours observed for the magnet pairs (*IP* appears on *F*_*2*_ vs *d* curves and *F*_*4*_ < *F*_*1*_ or *F*_*4*_ < *0*).

Pair ID	Magnet 1 (Top)			Magnet 2 (Bottom)				*For N → S pairs Unusual Force: F*_*4*_ < *F*_*1*_* (Usually F*_*4 *_= *F*_*1*_*)*				*For N →***← N** *pairs Unusal Force: F*_*2*_ vs gap *d has an inflection point IP when d* < *IP, |F*_*2*_*|*↓ *as d* ↓*, F*_*2*_* may* > *0 (Normally F*_*2*_ < *0)*								
	*L*_*1*_ (mm)	*OD* (mm)	|*P*_*c1*_|	*L2* (mm)	*OD* (mm)	|*P*_*c2*_|	*P*_*c1*_/*P*_*c2*_	N55			N48SH and SmCo30	N55			N48SH			SmCo30		
								*F*_*1*_ (*N*)@ *d* = 0.1 mm	*F*_*4*_ (*N*)@ *d* = 0.1 mm	∆F%*		*F*_*2*_* (N)@ d* < 0.5 mm	*F*_*2*_* (N)@ IP*	∆*F*_*2*_%	*F*_*2*_* (N)@ d* < 0.5 mm	*F*_*2*_* @ IP*	∆*F*_*2*_%	*F*_*2*_* (N)@ d* < 0.5 mm	*F*_*2*_* (N)@ IP*	∆*F*_*2*_%*
1-A	2	4	1.41	2	32	0.13	10.8	0.8	− 0.6	175	N/A	1.0	− 0.10	1100	0.15	− 0.10	250	0.00	− 0.05	100
2-A	2	8	0.61				4.69	2.5	− 0.5	120		2.2	− 0.4	650	0.3	− 0.5	160	− 0.10	− 0.16	38
3-A	2	16	0.28				2.15	6.4	0.2	97		3.8	− 2.1	281	− 0.3	− 2.3	87			
A-A	2	32	0.13				1	61	61	0		− 39.4	N/A			N/A		− 14.9	N/A	
4-A	16	4	24				185	1.4	− 1.3	193		2.7	− 0.9	400	− 0.4	− 0.7	43	− 0.327	− 0.34	4.7
4-B				4	32	0.28	85.7	2.3	0.2	91		2.3	− 1.6	244	− 1.1	− 1.4	21			
4-C				8		0.61	39.3	3.7	3.7	0		− 1.0	− 3.2	69	− 2.5	− 2.7	7			
4-D				16		1.41	17	5.8	5.8	0		− 4.1	− 4.7	13	− 4.0	− 4.1	2	− 1.86	− 1.94	4.3

*A larger *ΔF* or *ΔF*_*2*_ indicates a greater localized demagnetization *LD*.

### Localized demagnetization ***LD*** plots and maps and the determined ***LD*** levels

**Figure 9:**
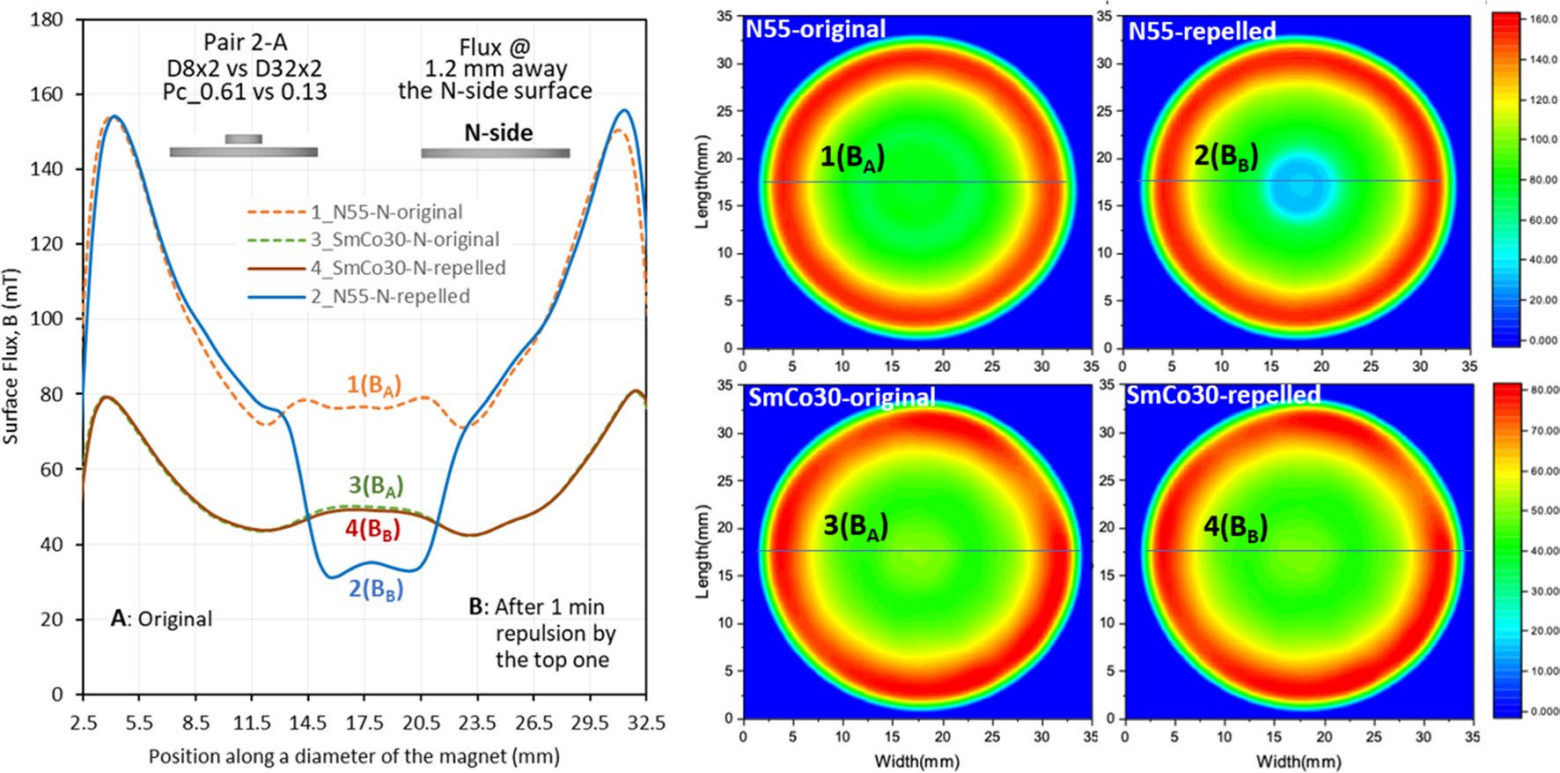
The surface field @ 1.2 mm above the N side of N55 and SmCo30 after pairing with *P*_*c*1_/*P*_*c2*_ = 4.69.

**Figure 10:**
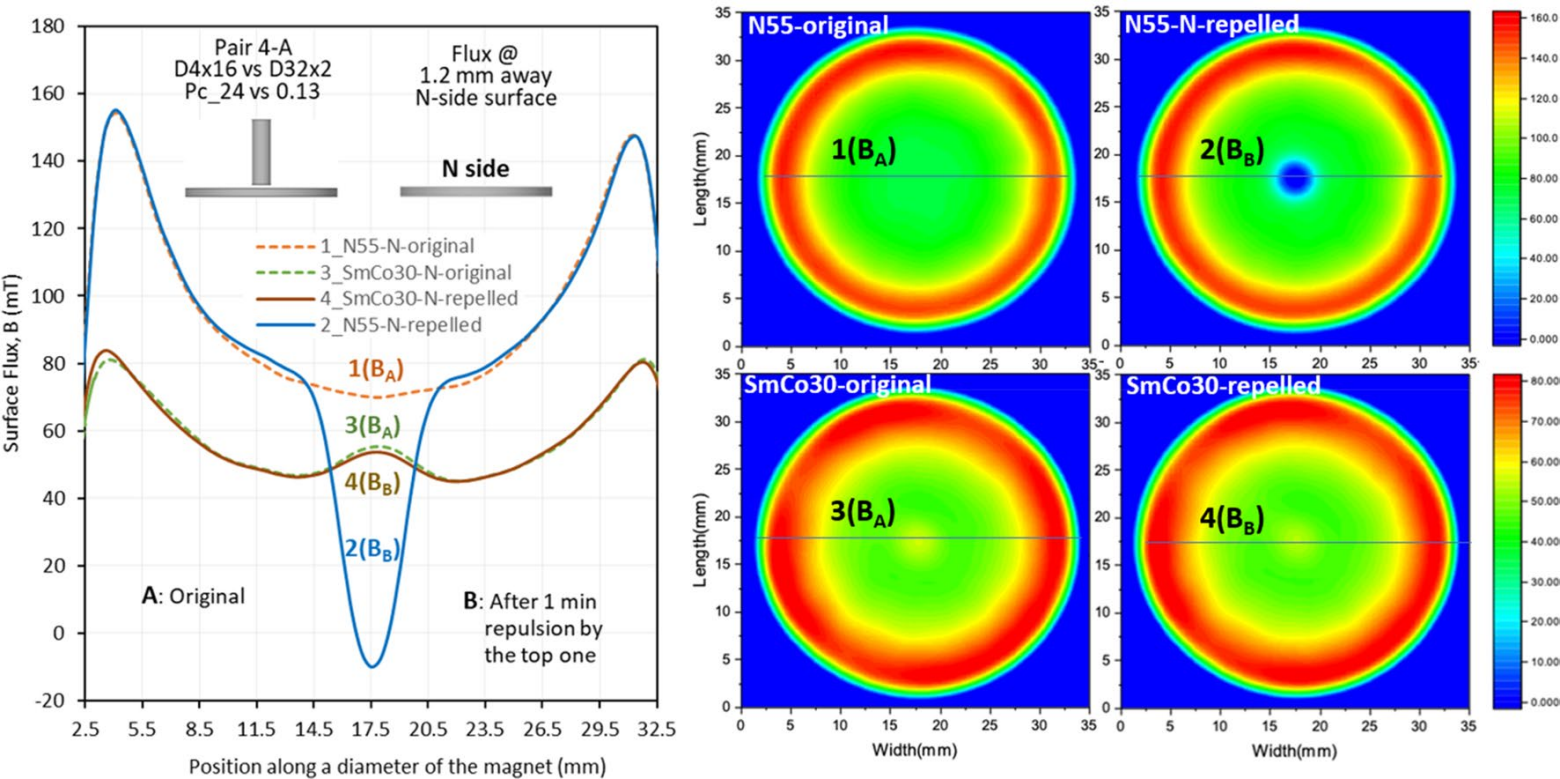
The surface field @1.2 mm above the N side of N55 and SmCo30 after pairing with *P*_*c*1_/*P*_*c2*_ = 185.

**Figure 11:**
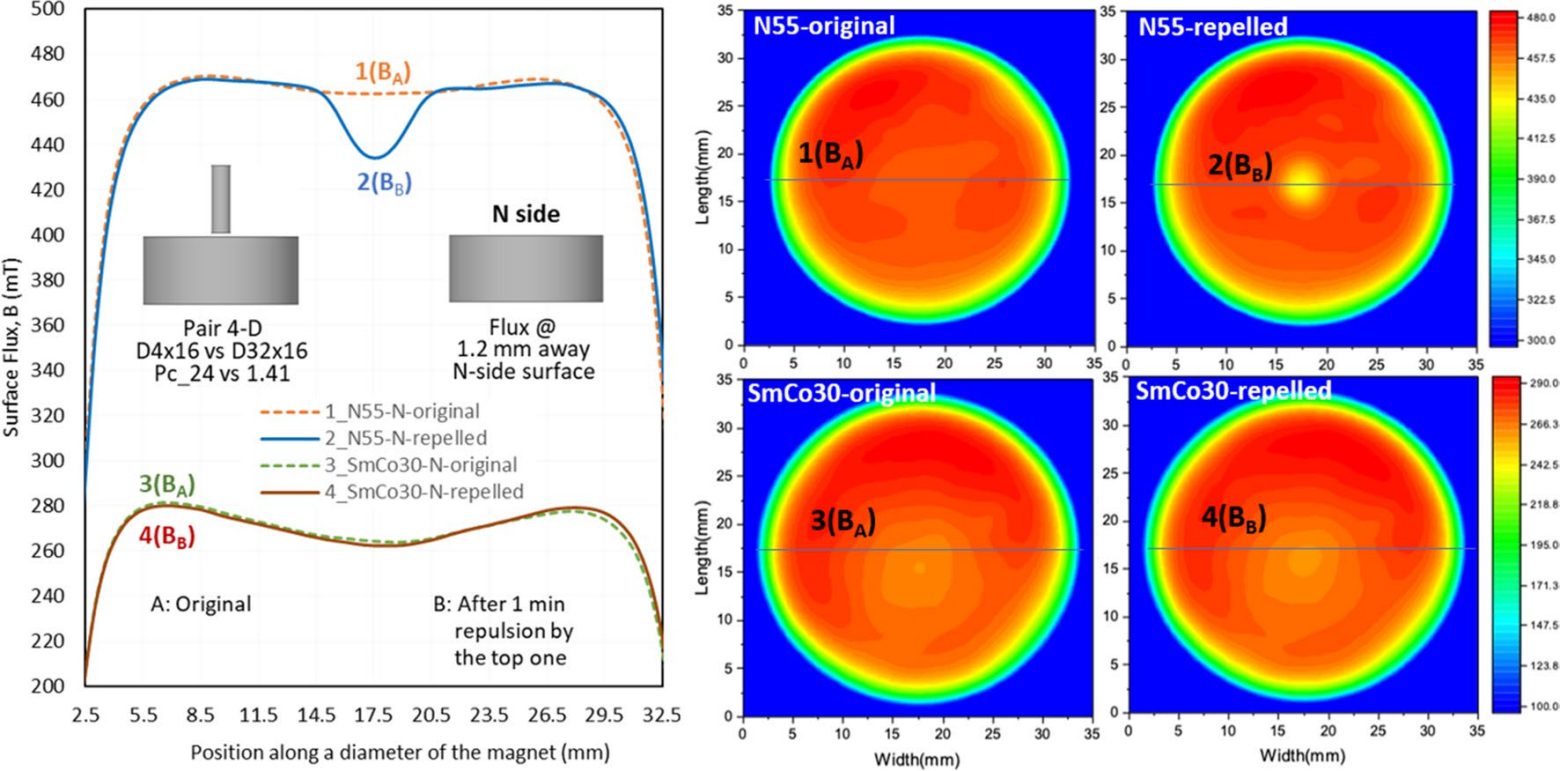
The surface field @ 1.2 mm above the N side of N55 and SmCo30 after pairing with *P*_*c*1_/*P*_*c2*_ = 17.

The *LD* can be visualized using a surface flux density test (the testing setup is illustrated in Fig. 3), from which the *LD* levels can be determined. Figures 9, 10 and 11 show the flux density at 1.2 mm above the N-side of the bottom magnets of N55 and SmCo30 with three *P*_*c*_ ratios of 4.69, 185, and 17, respectively. The 2D magnetic flux maps are shown on the right, and the curves of flux density vs position and the magnet size are shown on the left. The curves of flux density vs position along a diameter are curves 1 and 2 for N55, and curves 3 and 4 for SmCo30. Curves 1 and 3 are the original fluxes labelled “A”, corresponding to Step 1-Scan A in Fig. 3. Curves 2 and 4 are the fluxes after 1 minute of repulsion by the top magnet, labelled “B”, corresponding to Step 2-Scan B in Fig. 3. The flux densities at the surface of the magnets with *OD* = 32, as well as the *LD* values (*LD* = 100%*(*B*_B_ − *B*_A_)/*B*_A_) for various pairs are summarized in Table 3. Figure 9 shows pair 2-A with *P*_*c1*_*/P*_*c2*_ = 4.69, in which N55 has a larger *LD* (− 55%) at the center of curve_2, comparing to original curve_1, while SmCo30 has a small *LD* (− 2.1%). Figure 10 shows the pair 4-A with *P*_*c1*_*/P*_*c2*_ = *185*, and N55 has a huge *LD* with its polarity being totally reversed at the center (− 114%) of curve_2, while SmCo30 has a small *LD* (− 0.44%). Figure 11 shows pair 4-D with *P*_*c1*_*/P*_*c2*_ = *17*, and N55 has *LD* = − 6.1% while SmCo30 has *LD* = 0.64%. In general, *LD* on the N-side is higher than that on the S-side. When the *P*_*c1*_*/P*_*c2*_ ratio = 1 for the A–A pairs, the *LD* for both N55 and SmCo30 is approximately “0”, and the tiny difference is within the measurement error. Since the flux density was tested at 1.2 mm above the surface, the actual *LD* should be higher than the levels described in this section.

**Table 3:** The *LD* detected at 1.2 mm above the surface of bottom magnets (B_A_ – original testing after magnetization, B_B_ tested after repulsion on N **→← **N pairing).

Surface field, B (mT) @ 1.2 mm above	#2-A (*P*_*c1*_/*P*_*c2*_ = 4.69)			#4-A (*P*_*c1*_/*P*_*c2*_ = 185)			#4-D (*P*_*c1*_/*P*_*c2*_ = 17)			#A–A (*P*_*c1*_/*P*_*c2*_ = 1)		
	B_A_	B_B_	*LD* (%)	B_A_	B_A_	*LD* (%)	B_A_	B_A_	*LD* (%)	B_A_	B_A_	*LD* (%)
**N55**												
N-side	76.7	34.8	− 55	70.1	− 10.0	− 114	462	434	− 6.1	68.3	68	− 0.44
S-side	− 80.8	− 38.7	− 52	− 72.3	− 13.7	− 81	− 440	− 440	0.07	− 73.3	− 73.6	0.41
**SmCo30**												
N-side	50.3	49.24	2.1	55.2	53.6	− 2.9	264	263	− 0.64	48.1	48	− 0.21
S-side	− 49.2	− 48.2	− 2.0	− 59.7	− 58.4	− 2.2	− 288	− 287	− 0.31	− 47.9	− 47.8	− 0.21

### The effects of the linearity of B–H curves and the load-lines with working points

**Figure 12:**
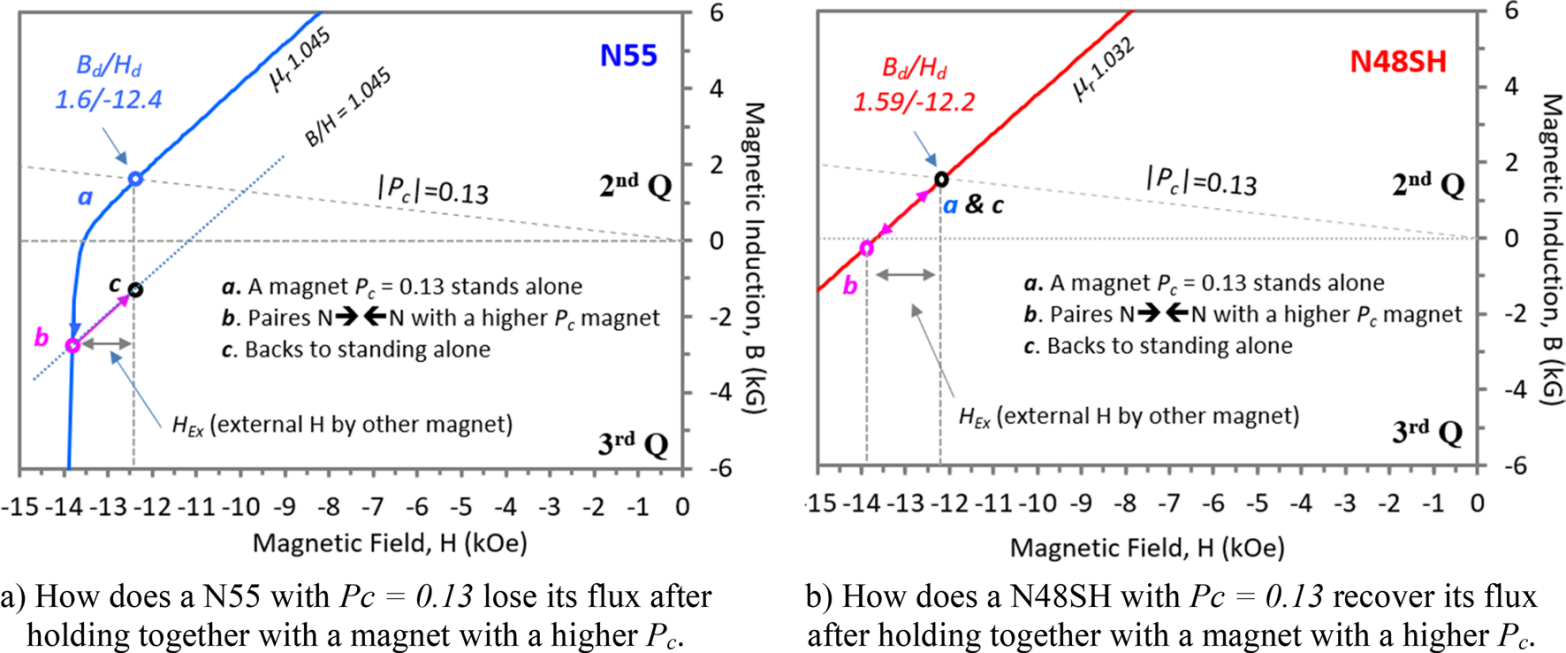
The B–H curves in the 2nd and part of the 3rd quadrant and the load-line function.

As shown in the previous sections, the *LD* level is related to the *P*_*c*_ ratio, and it is also linked to the linearity of the B–H curves in the 2nd and part of the 3rd quadrant. Figure 1 shows the B–H curves of N55, N48SH, and SmCo30, and the linearity limits (knee positions) of the B–H curves are 13 kOe, 21.4 kOe, and > 21.5 kOe, respectively. Among all three magnets, SmCo30 has the best linearity of the B–H curve. The small circles at the cross points of the load-lines and the B–H curves in Fig. 1 are the working points (*B*_*d*_*/H*_*d*_), which are listed in Table 4 for some magnets tested in this investigation. When two unequally sized magnets with different *P*_*c*_ pair with the like poles N **→← **N, the one with a lower *P*_*c*_, which has an internal self-demagnetization field *H*_*d*_, will be affected by an external field *H*_*ex*_ from the one with a higher *P*_*c*_. The total demagnetizing field is the sum of *H*_*d*_ and *H*_*ex*_ as shown in Fig. 12. If the sum exceeds the linearity limits, the B loss will be unrecoverable. Figure 12a shows how a N55 magnet with *P*_*c*_ = *0.13* loses its flux B after paring as N **→← **N with a magnet having a higher *P*_*c*_. The stand-alone magnet has operating point “*a*”, and it drops to point “*b*” while pairing with one having a higher *P*_*c*_. After the pair separates, the magnet can only return to point “*c*” as it needs to return to its stand-alone condition along the line parallel to its relative permeability *μ*_*r*_ = 1.045. This large B loss is due to the nonlinear B–H curve of N55, and the working point “*a”* of this magnet is very close to the knee of the B–H curve. On this aspect, N48SH and SmCo30 would not have such unrecoverable losses as long as the total demagnetizing field is less than 21.4 kOe and 21.5 kOe, respectively, where they are about to lose their linearity of their B–H curves (see Fig. 1 for the B–H curve details). Figure 12b shows how a N48SH with *P*_*c*_ = *0.13* maintains its flux B after paring N **→← **N with a magnet with a higher *P*_*c*_. This explains why N48SH and SmCo30 do not show the unusual behaviour of *F*_*4*_ < *F*_*1*_ at a small gap, and this recoverable *LD* is a unique characteristic to be utilized in some novel applications in the near future.

**Table 4:** Working points *B*_*d*_ and *H*_*d*_, for the following tested magnets (see  Fig. 1 for the B–H curves).

*P*_*c*_ or load-line |*B*_*d*_/*H*_*d*_|	Workind point, *B*_*d*_* (kG) *and* H*_*d*_* (kOe)*					
	N55		N48Sh		SmCo30	
	B_d_	H_d_	B_d_	H_d_	B_d_	H_d_
0.13	1.6	− 12.4	1.59	− 12.2	1.2	− 9.2
0.61	5.4	− 8.8	5.3	− 8.7	4.1	− 6.7
1.41	8.5	− 6.0	8.0	− 5.7	6.1	− 4.3
24	14.2	− 0.59	13.7	− 0.57	− 10.8	0.45

See Refs.^7,8,10,11^ for the concept used in the analysis. It is clear that the linearity of the B–H curve and the magnet’s load-line play important roles in the *LD* level and its recoverability after the magnet separates from the N **→← **N paring.

## Summary

A localized demagnetization (*LD*) is identified for unequally sized magnetic like poles as their *P*_*c*_ values are different, in which the pole with a higher *P*_*c*_ causes a *LD* to the pole with a lower *P*_*c*_. If the *LD* is large enough, the polarity of the localized area can be reversed, resulting in an attraction between two like poles in the *LD* area in a small gap. Two unusual behaviours are observed in this investigation.

1. An inflection point, *IP*, appears on the curves of the force vs gap for all the unequally sized like poles. The *IP* results in nonmonotonic curves, even an attraction for the like poles.
2. For some NdFeB magnets with a low coercivity and nonlinear B–H curve in the 2nd quadrant, a repulsion can occur for these unequally sized unlike poles after previously pairing with their like poles that left an unrecoverable *LD* and a reversed polarity area.

The unusual behaviours are not contradictory to the basic law of magnetism, and they are caused by the localized demagnetization *LD*. A higher *P*_*c*_ ratio results in a greater *LD*; the linearity of the B–H curves and the load-line also play important roles in the *LD* level. The *LD* level can be visualized and determined by mapping the surface flux.

## Discussion

The linearity of the B–H curve and the magnet’s load-line play important roles in the *LD* level and its recoverability after the magnet separates from the N **→← **N paring. For N55 with a nonlinear B–H curve, especially with small thickness, the *LD* is mostly unrecoverable. Similar to N55, Alnico magnets may also show the same unusual phenomena. For N48SH and SmCo30 magnets with linear B–H curves in the 2nd and part of the 3rd quadrant, the *LD* is mostly recoverable after the like pair is separated. If SmCo30 or N48SH pairs have a proper *P*_*c*_ ratio, the like poles can also appear attracting each other; since the *LD* is recoverable, some novel applications may be developed for utilizing these newly discovered unique characteristics.
